# HIV Testing Disruptions and Service Adaptations During the COVID-19 Pandemic: A Systematic Literature Review

**DOI:** 10.1007/s10461-023-04139-4

**Published:** 2023-08-07

**Authors:** William Mude, Hadijah Mwenyango, Robyn Preston, Catherine O’Mullan, Geraldine Vaughan, Gary Jones

**Affiliations:** 1 School of Health Medical and Applied Sciences, Central Queensland University, Cairns Campus, 42-52 Abbott Street & Shields Street, Cairns, QLD 4870 Australia; 2 School of Health & Social Care, Edinburgh Napier University, Sighthill Campus, Edinburgh, EH11 4BN UK; 3 School of Health Medical and Applied Sciences, Central Queensland University, Townsville Campus, Townsville, Australia; 4 School of Health Medical and Applied Sciences, Central Queensland University, Bundaberg Campus, Bundaberg, Australia; 5 School of Health Medical and Applied Sciences, Central Queensland University, Sydney Campus, Sydney, Australia; 6 Cohort Doctoral Studies Program, James Cook University, Cairns, Australia

**Keywords:** HIV testing, COVID-19, Service response, Impact, Utilisation, ART adherence, Patient care

## Abstract

Access to treatment and care in safe clinical settings improves people’s lives with HIV. The COVID-19 pandemic disrupted vital HIV programs and services, increasing the risk of adverse health outcomes for people with HIV and HIV transmission rates in the community. This systematic literature review provides a meta-analysis of HIV testing disruptions and a synthesis of HIV/AIDS services adapted during COVID-19. We searched scholarly databases from 01 January 2020 to 30 June 2022 using key terms on HIV testing rates and services during the COVID-19 pandemic. The process of how the included articles were identified, selected, appraised, and synthesised was reported according to the Preferred Reporting Items for Systematic Reviews and Meta-Analyses (PRISMA) guidelines. We included 17 articles that reported changes in HIV testing during the COVID-19 pandemic and 22 that reported adaptations in HIV/AIDS services. We found that HIV testing decreased by 37% during the search period because of the COVID-19 pandemic. Service providers adopted novel strategies to support remote service delivery by expanding community antiretroviral therapy dispensing, setting up primary care outreach points, and instituting multi-month dispensing services to sustain client care. Therefore, service providers and policymakers should explore alternative strategies to increase HIV testing rates impacted by COVID-19 and leverage funding to continue providing the identified adapted services.

The online version contains supplementary material available at 10.1007/s10461-023-04139-4.

## Introduction

People living with human immunodeficiency virus (HIV) and acquired immunodeficiency syndrome (AIDS) can experience an increasingly long and healthy quality of life. These advancements are associated with dramatic improvements in HIV/AIDS services, including but not limited to better access to testing, counselling, antiretroviral therapy (ART), and other social support. This scaling up of services has meant that many people living with HIV can now access treatment and care in a safe clinic environment. An important part of scaling up HIV/AIDS services globally includes the United States President’s Emergency Plan for AIDS Relief (PEPFAR) and The Global Fund, which support many countries in providing HIV/AIDS services [1]. These services have been instrumental in ensuring that people living with HIV in low-income countries have access to ART and regular testing to monitor viral load and psychosocial support with counselling and casework. Accordingly, the Joint United Nations Programme on HIV/AIDS (UNAIDS) updated its targets to ensure that 95% of people living with HIV know their status through testing, 95% receive ART, and 95% of people on ART have suppressed viral activities by 2030 [2]. However, in many settings, these services have been disrupted in a myriad of ways by the onset of the coronavirus disease (COVID-19) pandemic.

In January 2020, the World Health Organization (WHO) declared COVID-19 a public health emergency of international concern and designated it a pandemic in March 2020 [3, 4]. Countries responded by closing borders, introducing social distancing regulations, and grounding air travel worldwide [5–7] in what the International Monetary Fund (IMF) called the “Great Lockdown” [8]. The declaration of COVID-19 as a health emergency and its related intervention strategies disrupted the provision of many vital HIV/AIDS services [1]. Reports suggest that in areas with a high burden of HIV, COVID-19 disrupted HIV programme delivery and related healthcare services, especially among key communities [9]. Moreover, governments, notably in middle- and high-income countries, scaled back funding for international humanitarian and public health responses [10]. For example, The Global Humanitarian Assistance Report indicates that seven of the 20 largest donors reduced their humanitarian contribution by $3 billion [11]. It is claimed that these cutbacks were redirected to fund national responses to COVID-19, thereby negatively impacting the response to HIV [12]. These responses have exacerbated the profound systemic health inequities that have characterised the global response to HIV [13], compounding vulnerabilities and increasing the risk of new HIV transmissions. A report produced by UNAIDS suggests rises in HIV infections during the COVID-19 pandemic in regions where rates had previously been falling [14].

In 2021, approximately 38.4 million (33.9–43.8 million) people lived with HIV globally [15]. Of those, 28.7 million were on ART, all of whom require regular testing and clinical follow-up of their viral loads [15]. The disruptions to HIV/AIDS services across the globe during COVID-19 have put people living with HIV at risk of adverse health outcomes, including the development of opportunistic infections, drug resistance, comorbidities with other conditions, and increased mortality [1]. Concerningly, people living with HIV depend on ART to suppress their viral loads. Therefore, maintaining ART uptake is vital to their overall health and for preventing HIV transmission [16, 17]. Without access to essential treatment such as ART, rates of community HIV transmission will continue to increase [18]. Furthermore, modelling undertaken by Hogan et al. (2020) indicates that disruption in ART in HIV-endemic countries will lead to a 10% increase in HIV-related deaths (19). They argue that a lack of ongoing funding for HIV treatment and care for people living with HIV will make it impossible to deliver effective HIV public health responses, especially in low-income countries with limited health infrastructure [19].

To date, several systematic reviews have focused on compiling the health outcomes of COVID-19 among people living with HIV, the impact of COVID-19 on people living with HIV, and mental health-related consequences [20–24]. However, there is a gap in understanding the extent to which COVID-19 has disrupted HIV testing worldwide [25]. HIV testing is vital for people living with HIV because of its implications for identifying an index patient, initiating ART early, and preventing transmission. Therefore, this study reports a meta-analysis of HIV testing disruptions and a synthesis of HIV/AIDS service adjustments during COVID-19. We aimed to (1) determine the extent of HIV testing disruptions caused by COVID-19 and (2) examine existing literature to provide insights into how HIV/AIDS service providers responded to disruptions in HIV services caused by the COVID-19 pandemic. The information synthesised from this review aims to provide HIV healthcare professionals with practical strategies to engage people living with HIV/AIDS and inform policies to improve HIV treatment and care in future epidemic crises.

## Methods

This review follows the guidelines outlined in the Preferred Reporting for Systematic Reviews and Meta-analysis (PRISMA) document [26]. We searched Medline, Embase, ProQuest, EBSCOhost, Scopus, Web of Science, and Scopus from 01 January 2020 to 30 June 2022 using the search terms described in Supplementary Table 1. Table 1 below shows the inclusion and exclusion criteria.

**Table 1:** Inclusion and exclusion criteria

Inclusion criteria	Exclusion criteria
o Original studies	o Reviews only (rapid and systematic)
o Peer-reviewed articles	o Other non-peer-reviewed articles such as opinions, commentaries, editorials, blogs, letters, news articles, perspectives, and reports on websites
o Reported the adaptations of HIV/AIDS services due to COVID-19 or impacts on HIV testing during COVID-19	o Reported other outcomes or HIV/AIDS service adaptations or impacts on HIV testing due to other reasons
o Field reports published in a recognised scholarly journal	o Non-peer-reviewed field reports
o Published in English	o Published in other languages
o Published between January 2020 and June 2022	o Published before January 2020 or after June 2022

**Fig. 1:**
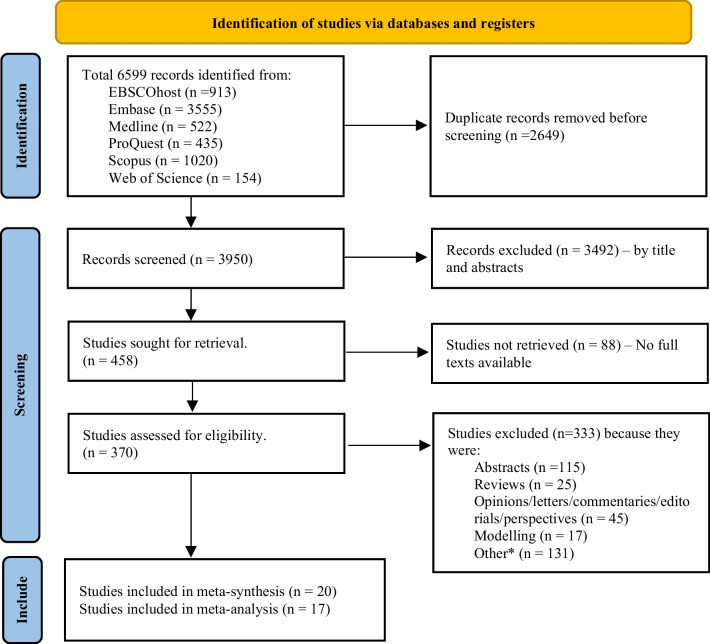
PRISMA Schema demonstrating screening and extraction steps. *These were studies published by various news sources, blogs, websites. Source: The PRISMA 2020 statement: an updated guideline for reporting systematic reviews (26)

Two researchers extracted the relevant studies based on the identified search terms, independently deduplicated the extracted studies and screened them by titles and abstracts to identify potential studies for further full-text screening. The two researchers discussed any discrepancies to reach a consensus and retained debated reports for further deliberation. The full-text screening was then independently performed on available included studies according to the eligibility criteria. Each step of the screenings and quality review was conducted by two assessors, followed by discussion and review by a third researcher where consensus was required. A PRISMA schema demonstrating these steps is provided in Fig. 1.

The studies that met the inclusion criteria were assessed for quality using the Joanna Briggs Institute (JBI) critical appraisal tool [27]. Predetermined information from included studies was documented in an Excel table, with data extracted on the authors, study type, aim, setting, study country, methods, publication year, study population, study period, and relevant findings (see Supplementary Tables 2 and 3) [28]. The included quantitative studies for the meta-analysis were assessed for risk of bias (cohort-type studies) using the Risk Of Bias In Non-randomized Studies—of Interventions (ROBINS-I) [29]. The risk of bias was categorised as either low risk (judged a low or moderate risk of bias in all domains) or high risk (severe or critical risk of bias in at least one part or where no information was provided in one or more key fields).

### Data Analysis

The outcome we measured in the meta-analysis was the HIV testing IRR before and during the COVID-19 pandemic. The IRR, log-transformed IRR (logeIRR), and standard error (SElogIRR) were calculated from reported HIV testing data for each included study before and during COVID-19 from January 2020 to June 2022, assuming a constant at-risk population over the study period. IRR was calculated by dividing the rate of HIV tests during COVID-19 by the rate of HIV tests before COVID-19. The SElogIRR was calculated by a formula $\sqrt{\left(\frac{1}{e\_Treat}\right)+\left(\frac{1}{e\_Control}\right)}$ as suggested by Rothman et al. [30]. The log-transformed IRR and the standard error were used to generate the forest plots using the random effect model. We found the change in HIV testing before and during the COVID-19 pandemic by calculating the percent decrease using the formula $\left(1-IRR\right)\times100\%$.

Sub-group analysis was performed for all the predicting factors by comparing IRR for study duration (less than or equal to 6 months, more than 6 months), study period (January–June 2020, after June 2020), study population (people living with HIV, general population), study method (cohorts, reports), study region (Africa, Europe, Americas, Asia and Pacific, multi-nations), risk of bias (low, high), study setting (sexual health clinic, general health facility, primary care clinic, or AIDS organisation centre), discussed lockdown (yes, no), world bank economic ranking (low- and lower-middle-income, upper-middle-income, high-income countries, multi-nations-uncategorised) and publication year (2021, 2022).

Multiple meta-regression was performed by imputing all the predictors into the model to predict the effect size and explain heterogeneity among the included studies at 95% confidence intervals. The regression was fitted using the REML method, and the Knapp and Hartung (knha) test was performed to determine statistical significance [31], which was indicated by an alpha of 0.05. All analyses were performed using R statistical software.

Publication bias was assessed by utilising a funnel plot and conducting Egger’s Test to determine the asymmetry of the funnel plot [32]. Also, a linear regression model of z-scores was regressed against the precision to determine the predicted effect size and the coefficient when the precision was zero [28]. Z-scores were calculated by dividing the reported decrease in HIV testing (IRR) in each study by their corresponding standard errors (seIRR). The precision score for each study was found by calculating the reciprocal of the standard error (seIRR). Z-scores were regressed against the precision scores using a linear model. Studies with no publication bias were expected to have their z-scores spread around zero [28]. The predicted effect was compared to the pooled result of our meta-analysis. Further, the Trim and Fill Method was used to correct for publication bias, and its pooled effect was compared to the pooled effect found before correcting for bias [33].

For the synthesis, we synthesised findings of the literature related to adaptations of HIV services by HIV/AIDS service providers during the COVID-19 pandemic, including community-based organisations, sexual health clinics, primary care clinics, and hospitals.

## Results

Thirty-seven documents were included in our study. Seventeen papers [25, 34–49] were included in the meta-analysis related to HIV testing, see Supplementary Table 2. For the meta-synthesis related to HIV service adaptations, 20 papers [50–69] were included, see Supplementary Table 3.

### HIV Testing

**Fig. 2:**
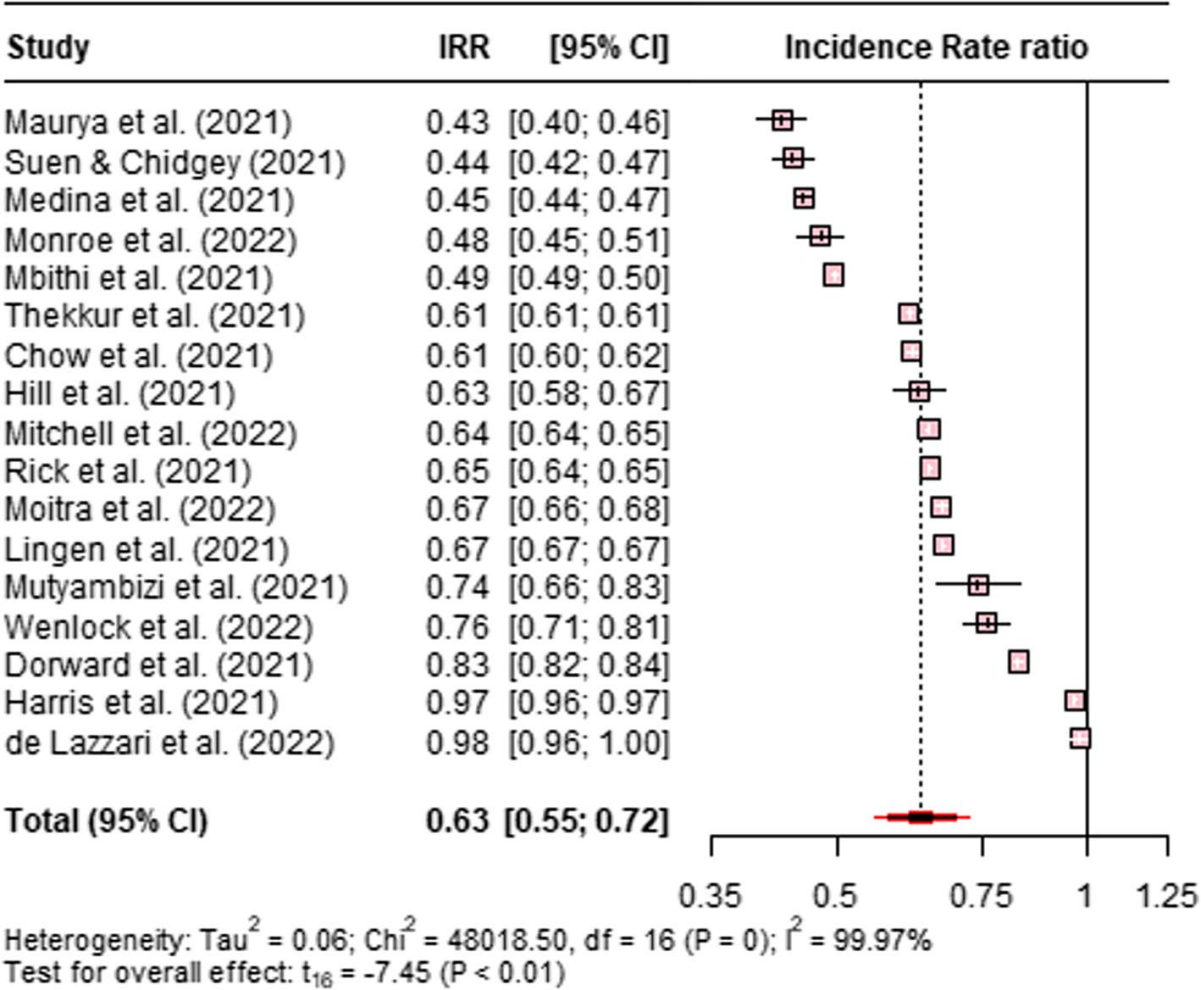
Forest plot for HIV testing IRR before and during the COVID-19 pandemic

We identified 17 papers that reported HIV testing during the COVID-19 pandemic. Six papers reported HIV testing in Africa, four in the Americas, four in Asia and the Pacific, and three in Europe. Of these 17 papers, 14 were cohort studies, and 3 were reports. Five articles were published in 2022, and the remaining in 2021. Figure 2 shows the pooled IRR using the random effects model for HIV testing before and during the COVID-19 pandemic. This finding shows a 37% decrease (IRR 0.63; 95% CI 0.55–0.72) in overall HIV testing during COVID-19 from January 2020 to June 2022. The t-test for the overall effect was significant, t = − 7.45, p < 0.05. However, our regression analysis shows that for every additional increase in HIV testing before the pandemic in 2019, there was a predicted 32% reduction in HIV testing during the pandemic, and this difference was statistically significant, t = 21.215, p < 0.05. See the section on publication bias for further information.

### Sub-group Analysis for HIV Testing

**Fig. 3:**
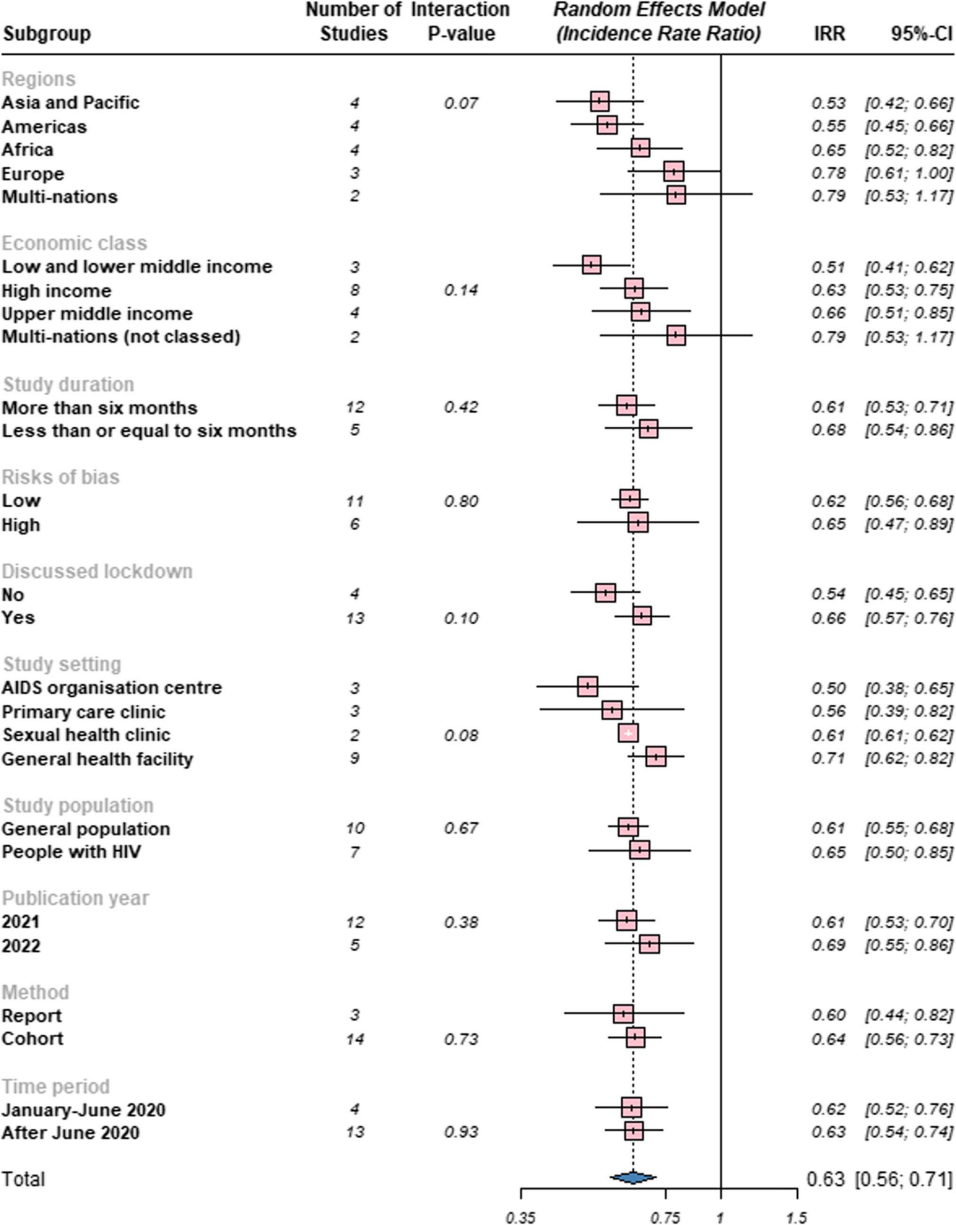
Forest plot for pooled sub-groups HIV testing IRR before and during the COVID-19 pandemic

Figure 3 shows the sub-group analysis for HIV testing IRR before and during the COVID-19 pandemic by region, economic class, study duration, risk of bias, whether the study discussed lockdown, study setting, study population, method, publication year, and time period. For further information on the forest plots for these sub-groups with their corresponding studies and Chi-square tests, see Supplementary Figs. A–J. When analysed by regions, HIV testing decreased by 47% (IRR 0.53; 95% CI 0.42–0.66) in Asia and the Pacific, 45% (IRR 0.55; 95% CI 0.45–0.66) in the Americas, 35% (IRR 0.65; 95% CI 0.52–0.82) in Africa, and 22% (IRR 0.78; 95% CI 0.61–1.00) in Europe. Analysis by economic class sub-group shows that HIV testing declined by 49% (IRR 0.51; 95% CI 0.41–0.62) in low and lower-middle-income countries, 37% (IRR 0.63; 95% CI 0.53–0.75) in high-income countries, 34% (IRR 0.66; 95% CI 0.51–0.85) in upper-middle-income countries, and 21% (IRR 0.79; 95% CI 0.53–1.17) in multi-nations (not classed).

Sub-group analysis by study duration found that HIV testing decreased by 39% (IRR 0.61; 95% CI 0.53–0.71) for studies conducted more than 6 months and 32% (IRR 0.68; 95% CI 0.54–0.86) for studies conducted for less than or equal to 6 months. Risk of bias sub-group analysis showed that HIV testing decreased by 38% (IRR 0.62; 95% CI 0.56–0.68) for studies deemed to have a low risk of bias and 35% (IRR 0.65; 95% CI 0.47–0.89) for studies assessed to have a high risk of bias. HIV testing rate decreased by 46% (IRR 0.54; 95% CI 0.45–0.65) in studies that did not discuss lockdown and 34% (IRR 0.66; 95% CI 0.57–0.76) in studies that examined lockdown.

Analysis by study settings showed that HIV testing decreased by 50% (IRR 0.50; 95% CI 0.38–0.65) in AIDS organisation centres, 44% (IRR 0.56; 95% CI 0.39–0.82) in primary care clinics, 39% (IRR 0.61; 95% CI 0.61–0.62) in sexual health clinics, and 29% (IRR 0.71; 95% CI 0.62–0.82) in general health facilities (e.g., hospitals). In the sub-group analysis for the study population, HIV testing decreased by 39% (IRR 0.61; 95% CI 0.55–0.68) in the general population and 35% (IRR 0.65; 95% CI 0.50–0.85) in people with HIV.

When analysed by year of publication, HIV testing dropped by 39% (IRR 0.61; 95% CI 0.53–0.70) for studies published in 2021 and 31% (IRR 0.69; 95% CI 0.55–0.86) for studies published in 2022. For study method sub-group analysis, the analysis indicates that HIV testing decreased by 40% (IRR 0.60; 95% CI 0.44–0.82) for studies categorised as “reports” and 36% (IRR 0.64; 95% CI 0.56–0.73) for studies grouped as “cohort”. In the sub-group analysis for Time Period, the study finds HIV testing decreased by 38% (IRR 0.62; 95% CI 0.52–0.76) for data collected between January and June 2020 and 37% (IRR 0.63; 95% CI 0.54–0.74) for data collected after June 2020.

### Multiple Meta-regression of Sub-group Analysis

Table 2 shows the results of the multiple meta-regression analysis of HIV testing IRR before and during the COVID-19 pandemic for sub-groups. Breakdown by region shows that during the study period, HIV testing was 21% (IRR 0.79; 95% CI 0.62–1.01) lower in the Americas, 22% (IRR 0.78; 95% CI 0.61–0.99) lower in Asia and the Pacific, and 12% (IRR 0.88; 95% CI 0.69–1.12) lower in Africa than in Europe. The difference in HIV testing rate between Asia and the Pacific, and Europe was statistically significant, t = − 2.188, p < 0.05.

**Table 2:** Multiple meta-regression for pooled sub-groups HIV testing IRR before and during the COVID-19 pandemic

Factors	Multiple meta-regression		
	IRR	t-test	p-value
Regions			
Europe (ref)	–	–	–
Americas	0.79 (0.62; 1.01)	− 2.049	0.063
Asia and Pacific	0.78 (0.61; 0.99)	− 2.188	0.049**
Africa	0.88 (0.69; 1.12)	− 1.154	0.271
Multi-nations	1.01 (0.76; 1.35)	0.092	0.928
Economic class			
High income (ref)	–	–	–
Upper middle income	1.02 (0.82; 1.27)	0.190	0.852
Low and lower middle income	0.87 (0.69; 1.10)	− 1.276	0.224
Multinational (not classed)	1.17 (0.89; 1.54)	1.209	0.248
Study duration			
More than 6 months (ref)	–	–	–
Less than or equal to 6 months	1.08 (0.89; 1.30)	0.802	0.435
Risk of bias			
High (ref)	–	–	–
Low	0.94 (0.78; 1.13)	− 0.756	0.461
Discussed lockdown			
No (ref)	–	–	–
Yes	1.14 (0.93; 1.39)	1.385	0.186
Study setting			
General health facilities (ref)	–	–	–
AIDS organisation centres	0.80 (0.64; 1.01)	− 2.065	0.060
Primary care clinics	0.87 (0.69; 1.09)	− 1.305	0.214
Sexual health clinics	0.90 (0.69; 1.18)	− 0.844	0.414
Study population			
People with HIV (ref)	–	–	–
General population	0.93 (0.78; 1.12)	− 0.819	0.426
Publication year			
2022 (ref)	–	–	–
2021	0.92 (0.76; 1.12)	-0.884	0.391
Method			
Cohort (ref)	–	–	–
Report	0.96 (0.76; 1.21)	-0.398	0.696
Study period			
After June 2020 (ref)	–	–	–
January–June 2020	0.98 (0.79; 1.21)	− 0.219	0.829

**Significant at 0.05 significance level

Despite the decrease not being statistically significant, economic class sub-group analysis shows that HIV testing decreased the most, by 13% (IRR 0.87; 95% CI 0.69–1.10), in low and lower-middle-income countries. There was a 2% (IRR 1.02; 95% CI 0.82–1.27) increase in HIV testing rate in upper-middle-income countries and 17% (IRR 1.17; 95% CI 0.89–1.54) increase in multi-nations (not classed) compared to in Europe, but these increases were not statistically significant.

Although not significant, there was a higher increase in HIV testing in studies conducted for at most 6 months (IRR 1.08; 95% CI 0.89–1.30) than in studies performed for more than 6 months. HIV testing in studies deemed to have had a low risk of bias was 6% (IRR 0.94; 95% CI 0.78–1.13) lower than in studies assessed to have had a high risk of bias. Additionally, HIV testing in studies that discussed lockdown due to COVID-19 was 14% (IRR 1.14; 95% CI 0.93–1.39) higher than those that did not discuss lockdown.

AIDS organisation centres experienced a 20% (IRR 0.80; 95% CI 0.64–1.01) decrease in HIV testing rates, the highest among all the settings examined in this study. The HIV testing rate in the general population decreased by 7% (IRR 0.93; 95% CI 0.78–1.12) more than in people with HIV during the COVID-19 pandemic in the studies reviewed. Likewise, the HIV testing rate reduced by 8% (IRR 0.92; 95% CI 0.76–1.12) more in studies published in 2021 than in studies published in 2022. Analysis by study methods shows that the HIV testing rate was 4% (IRR 0.96; 95% CI 0.76–1.21) lower in articles that were categorised as “reports” than in articles categorised as “cohorts”. In articles reporting findings conducted between January to June 2020, the HIV testing rate was 2% (IRR 0.98; 95% CI 0.79–1.21) lower than in articles reporting findings conducted after June 2020.

### Publication Bias

**Fig. 4:**
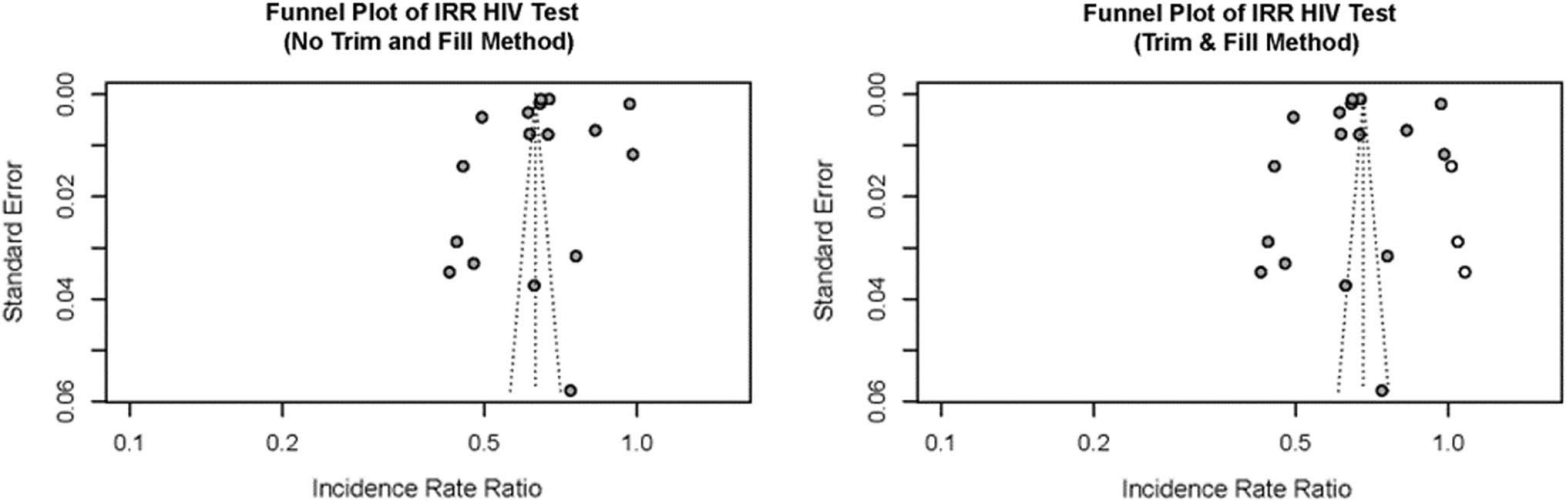
Funnel plot for HIV testing IRR before and during the COVID-19 pandemic with “no Trim and Fill” and with “Trim and Fill” method

The results to assess for publication bias by funnel plot suggested the presence of publication bias (Fig. 4). But Egger’s test did not indicate the presence of funnel plot asymmetry, t = 0.08, p = 0.935.

A linear regression model of z-scores against the precision found that when precision was zero, the intercept was 1.12 (t = 0.083, p = 0.935), which supports Eggert’s test of lack of publication bias. The predicted effect size of IRR was 0.68 (t = 21.215, p < 0.05), showing a predicted 32% decrease in HIV testing. Also, the Trim and Fill correction found a reduction of 32% (IRR 0.68; 95% CI 0.59–0.78) in HIV testing, which is slightly lower than the 37% decrease observed in the finding with no Trim and Fill Method (IRR 0.63; 95% CI 0.55–0.72).

### HIV/AIDS Service Adaptations During COVID-19

**Fig. 5:**
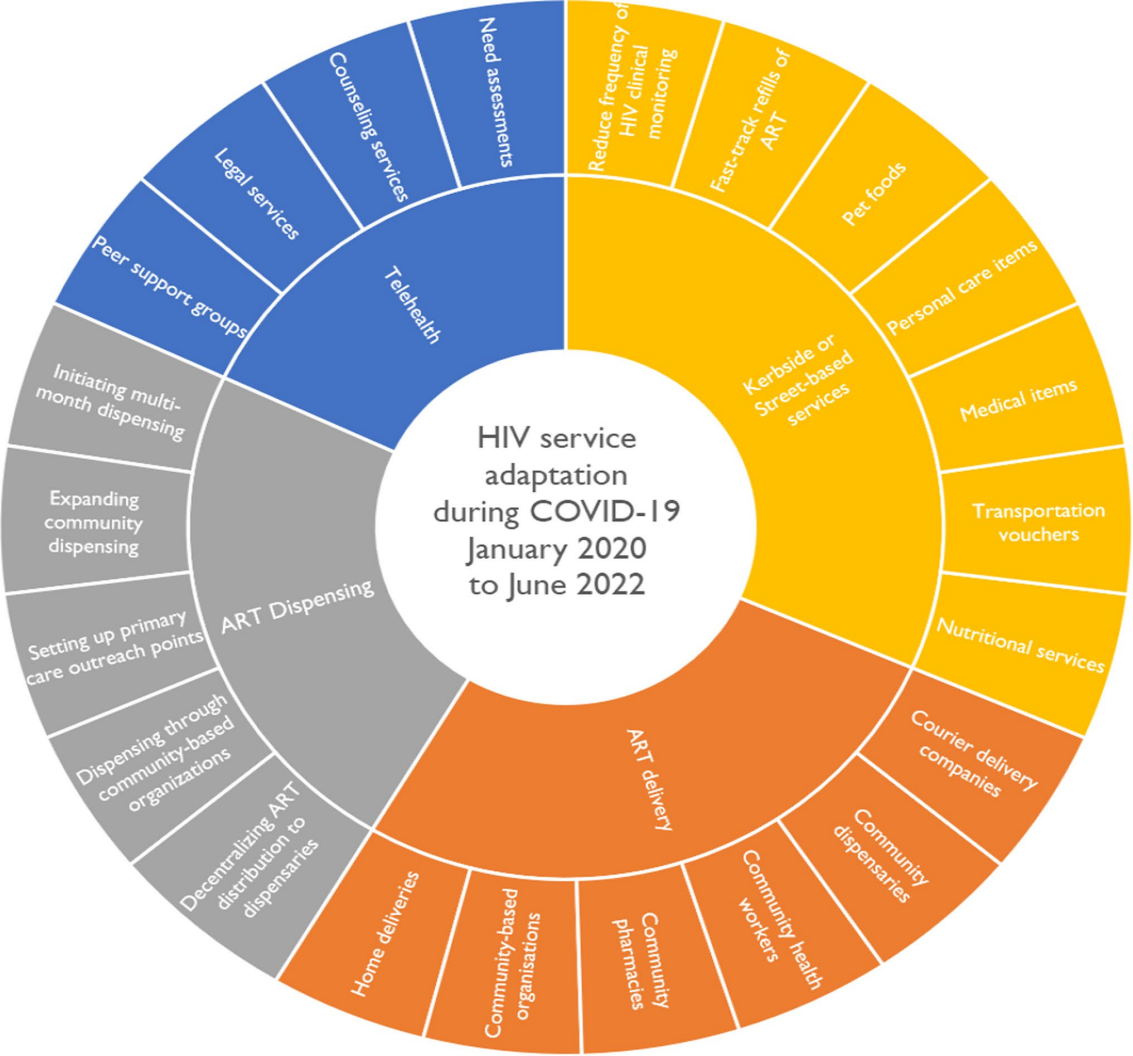
The mandala of HIV service adaptations during the COVID-19 pandemic

There were 22 reports included in the synthesis of health service adaptations, see Supplementary Table 3. Despite the profound disruptions caused by COVID-19, HIV/AIDS service providers were able to adapt services and respond to the myriad of challenges presented. HIV/AIDS service adaptation has been categorised into four key areas (Fig. 5), namely (i) Telehealth; (ii) Kerbside or street-based services; (iii) ART delivery; and (iv) ART dispensing.

### Telehealth

Service providers, particularly those in countries with high-level internet access, relied upon remote service delivery to conduct needs assessments and provide legal, counselling and support group services. Eleven studies in total reported providing HIV treatment and care during COVID-19 remotely through telehealth services such as telephone and videoconferencing. Five of the studies were from the United States of America (USA) [50, 52, 59, 60, 68] and one each from Zambia [63], Myanmar [62], Philippines [65], Indonesia [56], Tanzania [51], and Kenya [54]. These studies reported that providers completed the required paperwork, including conducting needs assessments electronically, either through e-mail or over the phone, to minimise in-person contact with their clients.

### Kerbside (Street-Based) Services

HIV/AIDS services are often tasked with addressing the social determinants of health (particularly housing and social support) as well as providing clinical care. However, it was impossible to provide such services remotely, especially those requiring in-person support. Four studies [53, 55, 58, 59] reported that people living with HIV requiring in-person support were provided with kerbside or street-based services and home delivery while limiting interaction. Kay and Musgrove [59] reported providing kerbside services in the USA, which included supporting clients with supplies such as food provisions, vouchers, pet food, and medical and personal care items. To minimise COVID-19 transmission, kerbside service staff loaded these supplies into the client’s car while limiting personal interaction. One study from Namibia described providing fast-track refills of ART for clients without having them enter the facility [58].

### ART Delivery

In addition to the kerbside service options described above, eleven studies reported adapting HIV Programme during COVID-19 by providing ART delivery services. Service providers utilised different delivery approaches to overcome barriers to clients accessing ART during the COVID-19 pandemic, ensuring people on ART stayed supplied with their medications. For example, HIV service providers delivered ART to community dispensaries, community health centres, community pharmacies, and other community-based organisations [54, 57, 61, 65, 66]. Several services utilised their existing network of community-based health workers to deliver ART [52, 55]. Others used mail and courier delivery companies or delivery vans to transport ART with discreet packaging [58, 64, 67, 69]. Home delivery was widely accepted and offered safe and sustained access to lifesaving ART treatment. Hoke et al. for example, reported that home delivery services were used by 51% of clients in Nigeria, 19% of clients in Indonesia, 21% of clients in Nepal, and 26% of clients in Laos during the study period [57]. In Botswana, 84% of HIV clients accepted the home delivery of ART through a courier service, which resulted in the delivery of 91% of successful ART refills [61]. Many service providers reported home deliveries in Asia (Indonesia, Laos, Nepal, India, and China) and Sub-Saharan Africa (Namibia, Nigeria, Uganda, Kenya, and Botswana) [54, 57, 58, 61, 64–67, 69].

### ART Dispensing

This meta-synthesis of HIV/AIDS service adaptions during COVID-19 showed that service providers prioritised ensuring people living with HIV requiring treatment did not run out of ART. Thirteen studies reported on how HIV/AIDS organisations maintained access to ART during the pandemic by expanding their ART dispensing points. This included developing community ART dispensing strategies, setting up primary care outreach points where clients could collect their medications, decentralising ART distribution to dispensaries, dispensing through community-based organisations [52, 58, 61, 64, 65, 67], and expanding or initiating multi-month dispensing [51, 53, 54, 60, 62, 66, 69]. For example, HIV service providers in Uganda utilised community drug distribution points to take ART closer to clients and facilitate access [69]. Some HIV service providers initiated multi-month dispensing services for the first time [54, 66], while others extended their multi-month dispensing services [51, 53, 54, 58, 60, 62, 69], for example, from three to 6 months.

## Discussion

This study examined the extent of HIV testing disruptions and service adaptations during COVID-19. The review found several notable findings. Firstly, there has been a decrease in overall HIV testing since the onset of the COVID-19 pandemic. Secondly, although we could not identify the source of heterogeneity in the pooled estimate of HIV testing rate through subgroup analyses, there were considerable differences in testing rates by region, especially between Europe and the Asia and Pacific region. Low- and lower-middle-income countries also reported a higher decrease in HIV testing rates than high-income countries. Likewise, the type of HIV testing facility that COVID-19 most impacted was AIDS organisation centres. Finally, providers adopted several strategies to deliver services during the COVID-19 pandemic, including using telehealth, introducing kerbside/street-based services, initiating or expanding ART delivery, and decentralising ART dispensing.

The study found that the overall HIV testing rate during COVID-19 from 01 to 2020 to 30 June 2022 was 37% lower than before the pandemic in 2019. The identified decrease in the HIV testing rate can be explained by the worldwide disruptions to HIV/AIDS service provision resulting from the COVID-19 pandemic. For example, a global cross-sectional study among men who have sex with men found a significant disruption to HIV testing throughout the pandemic [70]. Kay and Musgrove reported widespread closures of HIV services during the pandemic in the USA [59]. HIV facility-based testing disruptions and the temporary closure of drop-in testing centres have also been reported in Uganda [71], Kenya [72], and elsewhere [73]. The evidence also suggests that even when services were available, many people stayed away from HIV testing during COVID-19 because of the fear of COVID-19 exposure [74]. Our findings corroborate this evidence and demonstrate a widespread decrease in the uptake of HIV testing in different settings, with the HIV testing rate decreasing the most in HIV/AIDS organisation service centres. These findings show the extent to which the COVID-19 pandemic has impacted the HIV testing rate due to the profound disruptions affecting HIV/AIDS service delivery and fear of COVID-19 infection among people who were willing to access testing.

Differences in HIV testing rate reduction according to region were observed. The Asia and Pacific regions experienced the highest decrease in HIV testing rates, followed by the Americas, Africa, and Europe. However, a significant difference was observed only between Europe and Asia and the Pacific regions. These differences in HIV testing rate reductions were likely shaped by variations in lockdowns and regulations across and within different countries. For example, HIV service disruptions were significantly associated with lockdowns in China [75], while lockdowns prevented people living with HIV in Pakistan from accessing ART [76]. More stringent lockdowns in the Asia and Pacific regions might explain the highest decrease in HIV testing found in this study.

Similarly, our findings show that low and lower-middle-income countries experienced the highest decrease in HIV testing rates during COVID-19, followed by high-income countries, upper- and middle-income countries and multi-nations (not classed). Consistent with previous findings [77, 78], health systems in low-and middle-income countries may have been less equipped to manage competing health crises such as HIV and COVID-19. But the stratified multiple regression found that compared to high-income countries, there were no statistically significant differences in HIV testing rates during COVID-19 according to country economy ranking. This means the country economy ranking was not an important determinant of the HIV testing rate during the COVID-19 pandemic.

Our review also showed that studies conducted for more than 6 months reported a higher decrease in HIV testing rate than those performed for less than or equal to 6 months, corroborating the influence of study duration on the research findings [79]. Studies conducted for more than 6 months likely included many stay-at-home orders, which could have impacted the provision of and access to HIV testing. However, the difference in testing rate between the studies conducted for more than 6 months and those undertaken for less than or equal to 6 months were not statistically significant, suggesting study duration was not an important factor in determining the impact of COVID-19 on HIV testing rate during the pandemic.

Analysis by the risk of bias found that studies assessed as having a low risk of bias reported a higher decrease in HIV testing than studies deemed to have an increased risk of bias. This suggests that studies with a high risk of bias had underreported the impact of COVID-19 on HIV testing, while studies with a low risk of bias overreported the effect of the pandemic on HIV testing. However, the multiple meta-regression showed that the difference in HIV testing rate between studies assessed as having a low risk of bias and those deemed to have a high risk of bias were not statistically significant. The meta-analysis showed that studies that did not discuss lockdown reported a higher decrease in testing rate than those that did not. This suggests that studies that examined lockdown underestimated the impact of COVID-19 on the HIV testing rate and vice versa. But we found no significant differences in HIV testing rate between studies that discussed lockdown and those that did not.

This study also found that the health service setting most impacted by COVID-19 was AIDS organisation centres, which experienced the highest decrease in HIV testing rate, followed by primary care clinics, sexual health clinics and general health facilities (e.g., hospitals). However, with general health facilities considered as a reference, the multiple meta-regression found that the differences in the decrease of HIV testing rates among the different testing facilities were not statistically significant. This suggests that many people did not seek HIV testing during the pandemic, likely due to the fear of catching COVID-19 or the stringent lockdowns during COVID-19 [59, 71–74].

Regarding the study population, the general population experienced a higher decrease in HIV testing rate than people with HIV. But this difference was not statistically significant, indicating that COVID-19 impacted HIV testing rates equally in the general population and in people with HIV. Moreover, we found that the most considerable decrease in HIV testing was reported by studies published in 2021, but this was not significantly different from studies published in 2022. Similarly, studies conducted within the first 6 months of the global COVID-19 outbreak had the highest decrease in HIV testing rate. These results show that the pandemic impacted the HIV testing rate the most in the first half of 2020 when it started spreading worldwide. Testing moderately improved after June 2020, possibly because of adapting HIV/AIDS services by health care and service providers to suit the COVID-19 situation. These differences may also be explained by the workforce redeployment from HIV prevention and care to COVID-19 screening and testing during subsequent waves of the COVID-19 pandemic [46]. However, the difference was not statistically significant, suggesting that the study period did not influence the HIV testing rate. These findings indicate that the impact of COVID-19 on the HIV testing rate was statistically similar within the stratified factors. Additionally, studies categorised as “reports” reported more decrease in HIV testing rates than studies classified as “cohorts”. Most of these “reports” were studies from the field that could have reported particular cases, leading to the documented higher decrease in HIV testing rate [80], although this was not significantly different from the reported decreases by “cohorts”.

There was no evidence of publication bias, although the included studies seemed to have overestimated the overall effect of COVID-19 on HIV testing when analysed by the Trim and Fill method. The meta-analysis demonstrated a higher overall decrease in HIV testing before correcting, suggesting that the actual decline in HIV testing may be lower due to other factors not examined in this study. The linear regression model supports this assertion and found that the predicted decrease in the HIV testing rate was 32%.

The meta-synthesis of HIV service restructuring found that frontline professionals identified important and novel solutions as evidenced by accelerating alternative options, including telehealth, kerbside or street-based services, ART delivery and ART dispensing. For example, healthcare providers extended ART dispensation, introduced kerbside or street-based dispensation, and initiated distribution through community pharmacies to improve access to ART. Service providers responded to the disruption to HIV services by utilising telehealth for various purposes, including clinical consultations, running peer group activities, counselling and legal services and completing administrative tasks. There was acceptance of and increased use of remote service delivery. For example, McGinnis and Skanderson reported that 64% of clients received HIV services remotely in 2021 compared to 27% in 2019 [60]. However, an important consideration and caution in relation to in-home delivery is minimising the risk of compromising confidentiality and unwanted disclosure of HIV status [64]. The findings align with other studies employing rapid and flexible responses to ART delivery in emergencies [53, 81]. Furthermore, some of these responses have been proposed as sustainable healthcare improvements that can be adopted outside emergencies [78]. The necessary restructuring of services shows that while many of these alternatives may have been viewed as unmanageable before COVID-19, longer-term adoption can improve routine patient treatment and care for people with HIV. This finding also shows that when resources are brought together and service providers partner collaboratively with governments, industries, organisations and community stakeholders, it is possible to improve HIV/AIDS services and care.

However, our synthesis found that services relating to the prevention and testing of HIV were lacking in the service adaptations identified in this study. Perhaps this was due to the burden of clinical testing for COVID-19 within diagnostic services. For example, in Western Kenya, HIV testing resources (including personnel and equipment) were diverted to COVID-19 response strategies and testing of priority populations such as pregnant and breastfeeding women, leading to several months of delay in receiving results for viral load testing [74]. While there was no formal restructuring to facilitate HIV testing, several reports suggest an increase in the uptake of HIV self-testing kits occurred [1, 72, 82, 83]. In Kenya, some service providers introduced interventions in August 2020 to counteract low testing numbers associated with reduced testing services [72]. The scale-up of HIV self-test distribution as a sustainable and feasible approach has been suggested by several authors [84, 85]. However, we could not identify from our synthesis whether the reported decrease in HIV testing accounted for self-testing.

The identified decrease in HIV testing has several clinical, public health and policy implications, including the risk of delayed diagnosis and patients. Delayed ART initiation and associated increased risks of community transmission challenge achieving the WHO 2030 HIV/AIDS strategy targets. Therefore, achieving the WHO 95% testing target by 2030 will require more effort in closing the HIV testing gap caused by the pandemic. The identified gap also has implications for new HIV diagnoses and initiating ART. For example, a study in Italy found a decline of 31.2% in new HIV diagnoses in 2020 compared to 2019 [86]. In Japan, Ejima et al. reported an increase in the proportion of new HIV cases with an AIDS-related diagnosis from 24.4 to 36.2% in the first quarters of 2019 and 2020, respectively [87]. Our findings suggest that decreased HIV testing could result in delayed HIV diagnosis and missed opportunities for prompt ART initiation.

Some limitations in our study should be noted. Given the differences in COVID-19-related lockdowns and the different contexts in which the included studies were conducted, the data findings presented in this review should be interpreted with caution. Also, there are differences among the included reports as they used different data sources, and the periods in which the data were collected differed widely. More studies were published in 2021 than in 2022, which could lead to bias, although meta-regression found no significant difference according to publication year. Our synthesis could not identify if the reported decrease in HIV testing considered self-testing as part of the data. Therefore, this information needs consideration and caution when interpreting the findings presented in this meta-analysis. While international research was included in this review, we only considered studies published in English. This review may therefore be subject to some publication bias. Finally, our search only covered data through to June 2022. Given how rapidly this field is expanding, we anticipate that further evidence will become available.

## Conclusions

Our findings highlight the significant impact of the COVID-19 pandemic on HIV testing and service provision, with implications for policy and practice at a global, national, and organisation level. To close the identified gap COVID-19 has caused in HIV testing, governments and HIV support organisations must commit funds to scale up testing and strengthen meaningful collaboration and partnerships with communities and community-based organisations. The descriptions of novel responses to the impacts of COVID-19 on HIV testing shows the value of HIV/AIDS service providers continuing to leverage and extend their existing networks and community-based supports and work with peer groups and key population groups to promote HIV testing. Disruptions to HIV services and testing will likely continue post-COVID-19, particularly in low- and middle-income countries, due to the pressure of recovering from the pandemic’s devastations. Therefore, continuing to assess the pandemic’s impact on HIV testing and HIV/AIDS service provision for people living with HIV is essential. The significant effects of COVID-19 on health systems and global health equity calls for innovative responses that leverage funding and resources to better support the critical services required for HIV services amidst the long-term demands of responding to the COVID-19 pandemic. Future responses to infectious disease outbreaks should consider coordinated and flexible services to improve the accessibility and availability of HIV treatment and care for all people. Finally, we recommend any reporting of HIV testing in a future pandemic should include self-testing (or indicate if this is not accounted for) for improved reporting and accuracy.

### Supplementary Information

Below is the link to the electronic supplementary material.

Supplementary file1 (DOCX 214 KB)

Supplementary file2 (DOCX 15 KB)

Supplementary file3 (DOCX 24 KB)

Supplementary file4 (DOCX 29 KB)

## Data Availability

Not applicable.
