# Supplementary material for: HIV Testing Disruptions and Service Adaptations During the COVID-19 Pandemic: A Systematic Literature Review
Source: AIDS Behav. 2023 Aug 7;28(1):186–200. doi: 10.1007/s10461-023-04139-4 (PMC10803448; doi:10.1007/s10461-023-04139-4)

**(A)** Regions


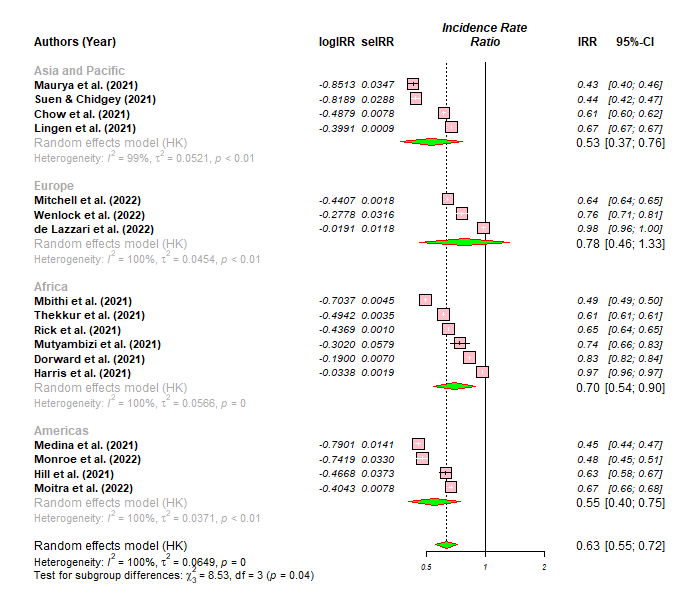


**(B)** Study duration (in months)


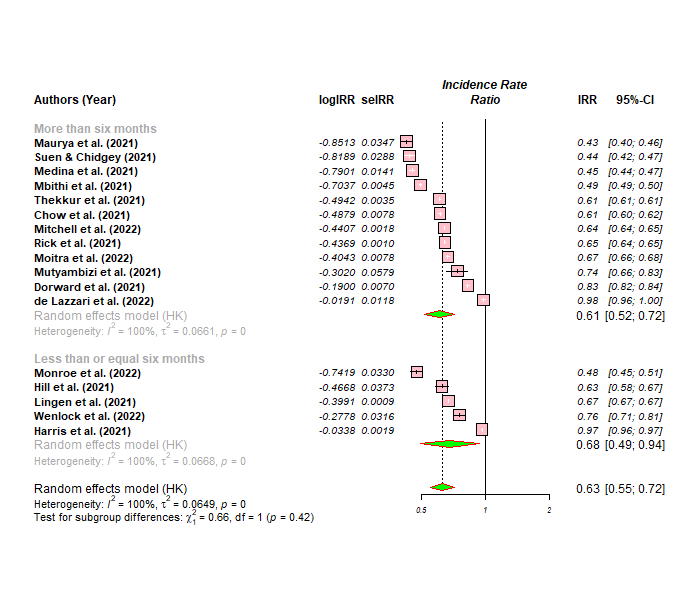


**(C)** Risk of bias


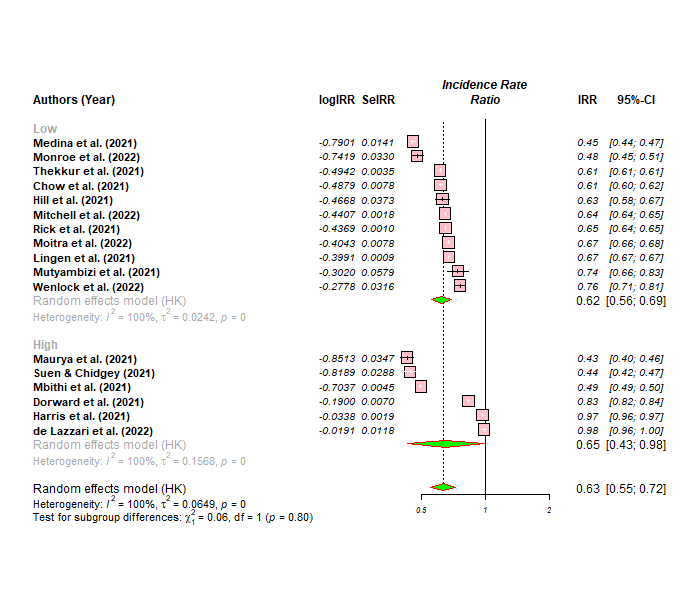


**(D)** Discussed lockdown


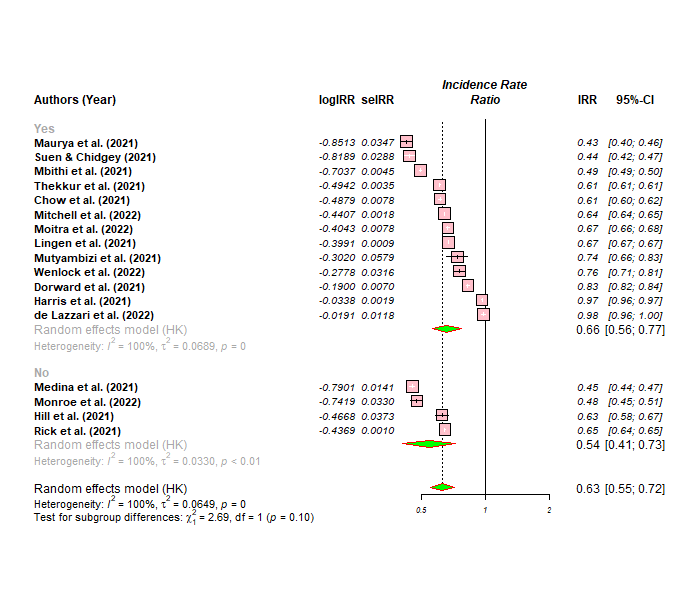


(E) Study Setting


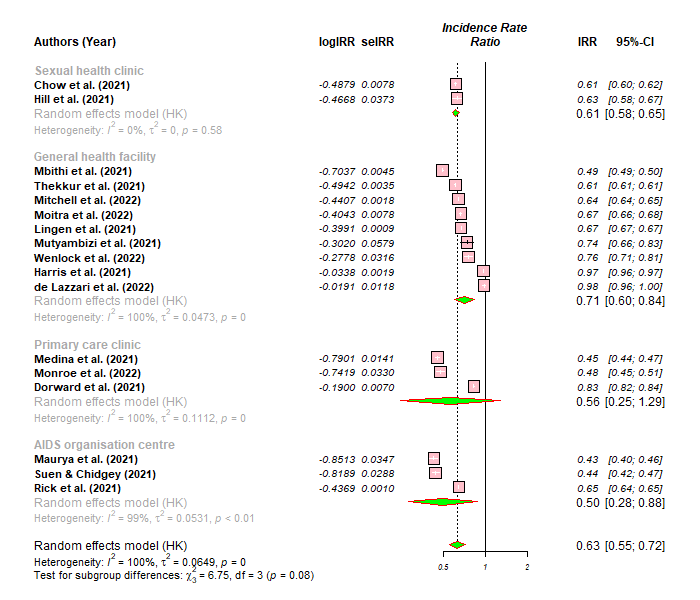


**(F)** Study Population


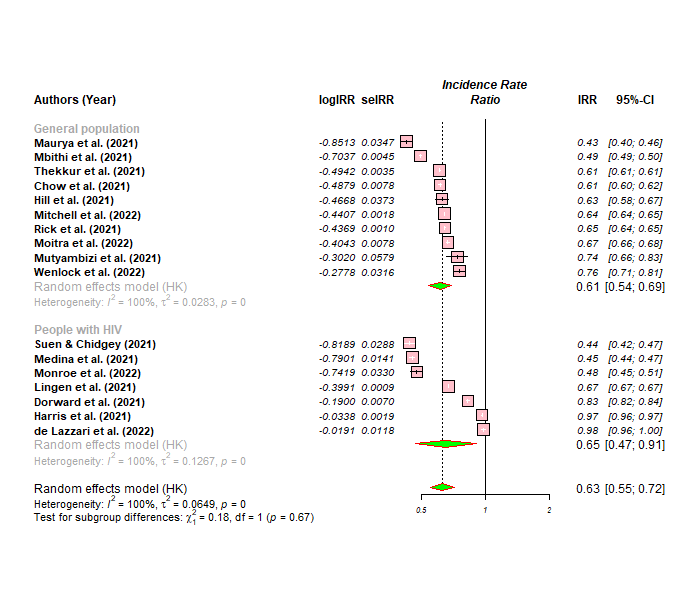


**(G)** Study Method


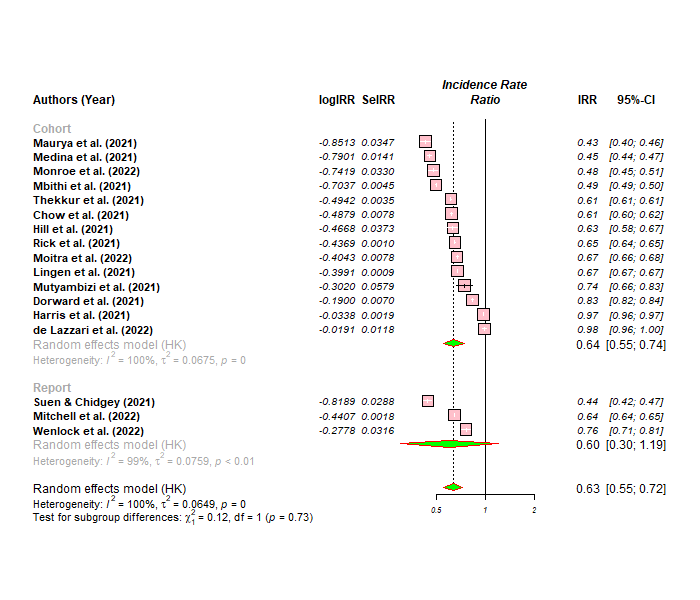


**(H)** Publication Year


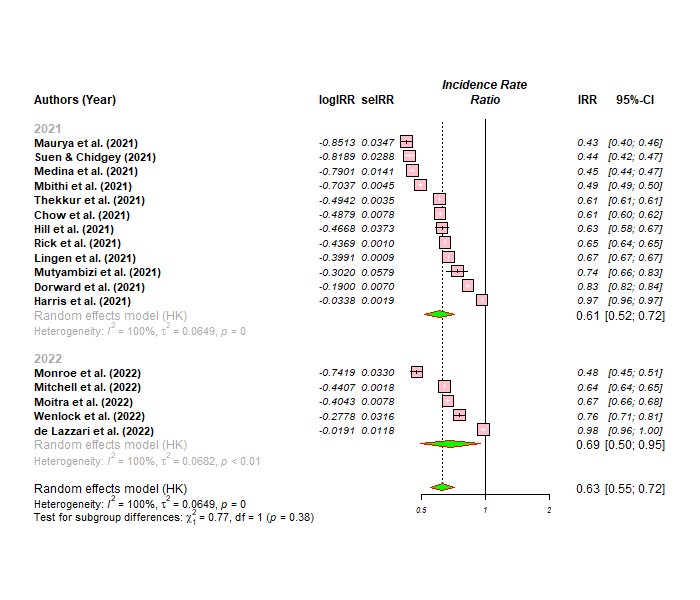


**(I)** Time period


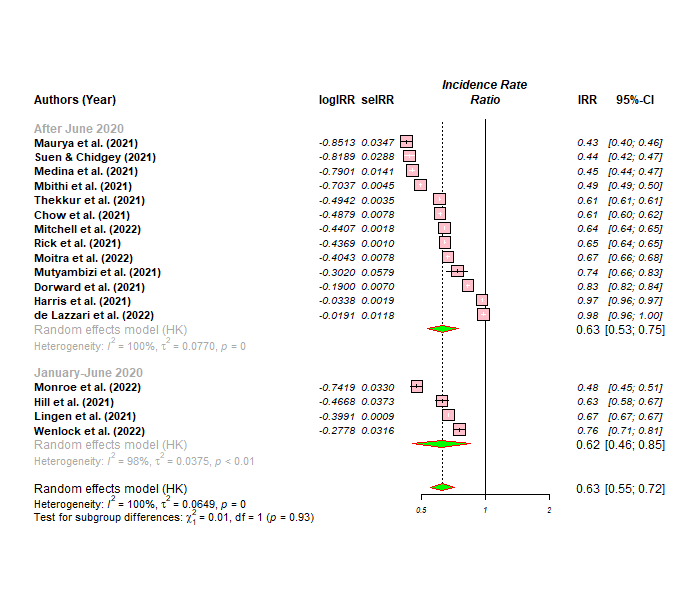


**(J)** Economic class


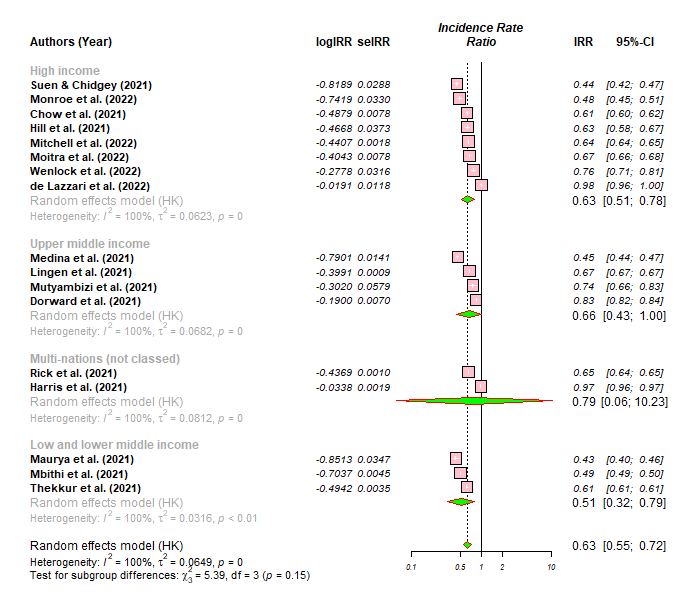

Supplement: Supplementary file 1 — Supplementary file1 (DOCX 214 KB) [file 10461_2023_4139_MOESM1_ESM.docx]
