# Supplementary material for: HIV Testing Disruptions and Service Adaptations During the COVID-19 Pandemic: A Systematic Literature Review
Source: AIDS Behav. 2023 Aug 7;28(1):186–200. doi: 10.1007/s10461-023-04139-4 (PMC10803448; doi:10.1007/s10461-023-04139-4)
Supplement: Supplementary file 2 — Supplementary file2 (DOCX 15 KB) [file 10461_2023_4139_MOESM2_ESM.docx]

**Supplementary table 1**: Search Terms

| **Key word** | **Search terms** |
| --- | --- |
| COVID-19 | “coronavirus” OR “nCoV*” OR “2019-nCoV” OR “COVID*” OR “SARS-CoV*” OR “COVID-19” OR “SARS Coronavirus 2 Infection” OR “COVID-19 Virus*” (title search) |
| HIV | “HIV” OR “human immunodeficiency virus*” OR “AIDS” OR “acquired immunodeficiency syndrome” (title search) |
| Service | “ART” OR “anti-retro viral therap*” OR “antiretroviral*” OR “care” OR “service*” OR “treatment” OR “test*” OR “screen*” OR “outreach*” OR “Access*” OR “PrEP uptake” OR “Prophylaxis” OR “healthcare” OR “time” OR “out-of-facility” OR “community-based” OR “walk-in” OR “home-based” OR “dispensing” OR “counseling” OR “delivery” OR “telehealth” OR “utilisation” OR “utilisation” OR “respon*” OR “reposition*” OR “implement*” OR “transition*” OR “adjust*” OR “introduc*” OR “initiat*” OR “disrupt*” OR “interrupt*” OR “increas*” OR “decreas*” OR “gain*” OR “loss*” OR “reduc*” OR “chang*” OR “impact*” OR “consequence*” OR “barrier*” OR “enabler*” OR “care experience*” OR “consequence*” OR “effect*” OR “engag*” (abstract search) |
