# Supplementary material for: HIV Testing Disruptions and Service Adaptations During the COVID-19 Pandemic: A Systematic Literature Review
Source: AIDS Behav. 2023 Aug 7;28(1):186–200. doi: 10.1007/s10461-023-04139-4 (PMC10803448; doi:10.1007/s10461-023-04139-4)
Supplement: Supplementary file 3 — Supplementary file3 (DOCX 24 KB) [file 10461_2023_4139_MOESM3_ESM.docx]

**Supplementary table 2:** Reports included in the meta-analysis of HIV testing disruptions.

| Author | Aim | Setting | Study country | Study method | Data type | Study population | Study period | Outcome | Finding |
| --- | --- | --- | --- | --- | --- | --- | --- | --- | --- |
| Chow et al. (2021) | Compare number of HIV post-exposure prophylaxis (PEP) prescriptions, HIV tests, and new HIV diagnoses during lockdowns | Sexual Health Centre in Melbourne and Sydney | Australia | Cohort data | Electronic medical records | General population | 2019-2020 | Weekly number of HIV tests and PEP prescriptions | 37% reduction in PEP prescriptions in Melbourne (1273 prescription in 2019 vs 796 in 2020; IRR=0.63, CI:0.57-0.68). 46% reduction in PEP prescriptions in Sydney (485 in 2019 vs 261 in 2020; IRR=0.54; CI:0.46-0.63). 41% reduction in HIV tests in Melbourne (31952 in 2019 vs 18894 in 2020; IRR=0.59, CI:0.58-0.60). 32% reduction in HIV tests in Sydney (11640 in 2019 vs 7868 in 2020; IRR=0.68; CI:0.66-0.70). 44% reduction in new HIV diagnoses in Melbourne (71 in 2019 vs 40 in 2020; IRR=0.56; cI:0.38-0.83). 47% reduction in new HIV diagnoses in Sydney (30 in 2019 vs 16 in 2020; IRR=0.53; CI:0.29-0.98). |
| de Lazzari et al. (2022) | Assess the impact of COVID-19 on prevention and care of HIV | Administrative hospital in Catalonia | Spain | Cohort data | Clinical data | People with HIV | March-December 2020 vs 2019 | The impact of COVID-19 on prevention and care of HIV | 28% reduction in HIV diagnosis. Scheduled visits reduced by 25%. 753 PEP visits in 2020 vs 1380 in 2019 (45% reduction, IRR = 0.55, CI; 0.5-0.6, p<0.001). 14349 tests in 2020 vs 14625 in 2019, (RR = 0.98, CI: 0.96-1.00, p=0.105). 143 new diagnoses in 2020 vs 199 in 2019 (28% reduction (IRR = 0.72, CI:0.58-0.89, p=0.003). 9830 outpatient visits in 2020 vs 13024 in 2019 (25% reduction; IRR=0.75, CI 0.74-0.77, p<0.001) |
| Dorward et al. (2021) | Measure the COVID-19 impact on testing and treatment | Primary care clinics in KwaZulu Natal | South Africa | Interrupted time series analysis | Routine HIV service delivery data from district health information system | People with HIV | 1 July 2018-31 July 2020 | HIV test per month, ART initiation per week, ART collection per week | 47.6% decrease in HIV testing (IRR=0.524, CI:0.446-0.615) (38911 vs 1997). ART initiation decreased by 46.2% (IRR=0.538; CI:0.459-0.630). ART collection visits IRR=0.932; CI:0.794-1.093). Increased missed ART collection visits (IRR=1.926; CI:1.585-2.341) |
| Harris et al. (2021) | Examine the effect of COVID-19 on HIV services | Health facilities | Sub-Saharan Africa | Report | Electronic medical records | People with HIV | October 2019-March 2020; and April-September 2020 | HIV testing, treatment | 3.7% increase in HIV testing (from 854880 to 930218). 4.2% increase in number of people on ART. 1% reduction in number of people initiating ART. |
| Hill, Anderson, and Lock (2021) | Number of pre-exposure prophylaxis (PrEP), HIV testing, and STI testing visits | Sexual health clinics in Arkansas, Missouri and Oklahoma | United States | Cohort data | Electronic medical records | Males | 1 March - 30 June 2020 | Visits for PrEP, HIV testing visits, and STIs testing visits | 3.9% increase in PrEP visits; 58.7% decrease in HIV testing (from 2452/12012 to 1013/7917); 7.5% reduction in HIV testing visits; and 7.6% decrease in STI testing |
| Lingen et al. (2021) | Evaluate the impact of COVID-19 on HIV care continuum | Jiangsu province | China | Cohort | Programmatic data | General population | Jan-March 2020 | HIV testing, new HIV diagnosis, positivity rate, ART initiation | HIV tests reduced from 11326388 in 2019 to 1875685 in 2020 (49% reduction by 919938); new diagnoses reduced from 2401 to 980; |
| Maurya, Sharma, Singh, Gautam, and Das (2021) | examine how COVID-19 impacted HIV services | integrated counselling centre | India | Service data | Records on HIV testing and diagnosis | General population | 2016-2020 | Number of HIV tests | Testing reduced by 57% (from 2743 in 2019 to 1182 in 2020), diagnoses of new infection reduced by 52% (from 572 in 2019 to 262 in 2020), client-initiated testing decreased by 48.8%, provider-initiated testing decreased by 60.3% |
| Mbithi et al. (2021) | Determine the impact of COVID-19 on HIV services | 18 health facilities in Nairobi | Kenya | Cohort | HIV service aggregate data | General population | March 2020-February 2021 | HIV-related parameters, diagnosis, and treatment | HIV testing reduced by 50.5% (from 150155 in 2019 to 74287 in 2020-2021), new diagnoses reduced by 30% (from 3819 in 2019 to 2673 in 2020); positivity rate increased by 1.1% (from 2.5 in 2019 to 3.6 in 2020), and ART initiation increased by 1.7% (from 91.2% to 92.9%) |
| Medina et al. (2021) | Describe the impact of COVID-19 on the diagnosis of HIV | Referral clinic and diagnostic laboratory hub for 13 HIV healthcare facilities | Guatemala | Cohort | Surveillance data | People with HIV | March to August 2019 and March-August 2020 | All patients diagnosed with HIV, and cases of opportunistic infections | 54.7% reduction in tests (16218 pre_COVID-19 to 7360 during COVID-19); new diagnoses reduced by 10.7% (216 cases in 2020 vs 242 cases in 2019) |
| Mitchell et al. (2022) | Assess the effects of COVID-19 on health outcomes for persons with STIs, HIV or hepatitis | Clinics and laboratories | England | Cohort | surveillance | General population | January - September 2019 and January - September 2020 | Testing for STIs and HIV | HIV testing at facilities reduced by 71% (from 95455 in 2019 to 22332 in 2020); number of tests for HIV was lower by 36% (768216 in 2019 vs 494433 in 2020); |
| Moitra et al. (2022) | Measure HIV testing and positivity rates during COVID 19 | Healthcare systems in metropolitan areas of several US regions | United States | Cohort | Electronic health records | General population | January – December 2019 and January – December 2020 | Number of HV tests per week, HIV positive rate per week | 40620 tests in 2019 vs 27112 in 2020; in total HIV tests reduced by 19.94%; HIV positivity test reduced by 15.84% |
| Monroe et al. (2022) | Examine the pandemics' impact on HIV care | Clinical sites in Washington DC | United States | Cohort | Electronic health records | People with HIV | March - June 2019 and March - June 2020 | Number of monthly HIV testing | 52.4% reduction in HIV testing (from 2839 in 2019 to 1352 in 2020), number of unique participants using service reduced by 23.5%, number of service use increased by 20.5% |
| Mutyambizi et al. (2021) | Measure impact of COVID-19 on HIV, TB and PMTCT health indicators | health facilities in Moponi District Municipality | South Africa | Cohort | Electronic records from district health information system | General population | April-December 2019 and April-December 2020 | Monthly HIV service indicators, ART indicators | 24% reduction in HIV service use; 26.2% reduction in HIV tests (from 22293 in 2019 vs 16450 in 2020); HIV positivity test reduced by 26.1% (from 702 in 2019 to 519 in 2020); ART initiation reduced by 247% (from 886 in 2019 vs 673 in 2020); ART retention reduced by 70% (659 in 2019 vs 198 in 2020) |
| Rick, Odoke, Hombergh, Benzaken, and Avelino-Silva (2021) | Compare HIV testing, percentages positive, in-person appointments, new enrolments in HIV care | AIDS Healthcare foundations | Multinational | Cohort | Health data from the AIDS Healthcare Foundation | General population | January-August 2019 and January-August 2020 | HIV testing, percentages positive, in-person appointments, new enrolments in HIV care | Created flexible program that was creative and relevant to the local context and situations e.g. MMD, telehealth, extended hours, community/postal/home delivery of ART, self-testing, community sample collection etc; number of HIV tests reduced by 35.4% (35.34-35.46) (from 2749320 in 2019 to 1776102 in 2020); HIV positivity increased by 9.52% (6.61-13.18); number of HIV service use reduced by 5.93% (5.91-5.96); number of new HIV enrolment in HIV care reduced by 38.19% (38.01-38.36) |
| Suen and Chidgey (2021) | Document strategies of service continuation and number of tests | AIDS Concern Hong Kong | Hong Kong | Cohort | Programmatic data | People with HIV | January -September 2019-2021 | Number of tests | Promoted self-testing; overall tests reduced by 40% in 2019 vs during COVID (2020-2021); tests reduced by 52.5% from 2019 to 2020 (from 4475 in 2019 to 2127 in 2020); tests reduced by 26.8% from 2019 to 2021 (from 4475 in 2019 to 3277 in 2021); tests increased by 54.1% (from 2127 in 2020 to 3277 in 2021) |
| Thekkur et al. (2021) | Measure the impact of COVID-19 on TB and HIV services in Lilongwe | Health facilities in Lilongwe | Malawi | Cohort | Routinely collected data | General population | March 2019-February 2020; March 2020-February 2021 | Number of testings, new positive testing, positivity rate | Testing for HIV reduced by 39% (from 210057 pre-COVID-19 to 128153 during COVID), new positive HIV diagnoses declined by 30.4% (from 7040 to 4900); positive rate increased by 0.4% (from 3.4 to 3.8); ART initiation declined by 1.4% (100% to 98.6%) |
| Wenlock et al. (2022) | Describe the impact of COVID-19 on HIV testing | Brighton and Hove | United Kingdom | Cohort | Electronic health records | General population | 2016-June 2020 | Monthly HIV testing | 19% reduction in tests (from 2321 to 1758); test increased by 2% (from pre-COVID-19 to 2021; tests increased by 26% (from 2020 to 2021); new diagnoses reduced from 3.6 per month pre-COVID-19 to 1.2 in 2020, and 1.8 in 2021) |
