# Supplementary material for: HIV Testing Disruptions and Service Adaptations During the COVID-19 Pandemic: A Systematic Literature Review
Source: AIDS Behav. 2023 Aug 7;28(1):186–200. doi: 10.1007/s10461-023-04139-4 (PMC10803448; doi:10.1007/s10461-023-04139-4)
Supplement: Supplementary file 4 — Supplementary file4 (DOCX 29 KB) [file 10461_2023_4139_MOESM4_ESM.docx]

**Supplementary table 3:** Reports included in the meta-synthesis of HIV service adaptation.

| Author | Aim | Setting | Study country | Study method | Data type | Study population | Study period | Outcome | Finding |
| --- | --- | --- | --- | --- | --- | --- | --- | --- | --- |
| Auchus, Jaradeh, Tang, Marzan, and Boslett (2021) | To quantify the impact of telehealth on retention in care for a vulnerable HIV-infected population and to identify patient perspectives of telehealth and its effect on appointment attendance | HIV primary care clinic in urban San Francisco, California | United States of America | Quantitative study | Survey | Patients | June – July 2020 | The impact of telehealth on retention in care for a vulnerable HIV-infected population and to identify patient perspectives of telehealth and its effect on appointment attendance | Shifted patients to telemedicine due to the COVID-19 pandemic. Between 1 October 2019 and 16 March 2020, telehealth comprised 5.3% of total clinic appointments. But between 17 March 2020 and 30 June 2020, telehealth comprised 86.9% of all clinic appointments. |
| Bajaria and Abdul (2020) | Evaluates the current capacity of facilities and providers for HIV care and treatment services and their preparedness | Nationwide | Tanzania | Survey | Tanzania survey provision assessment survey | People living with HIV and healthcare workers | Not specified | Document capacity of facilities and providers for HIV care and treatment services and their preparedness | Training on COVID-19 precautions and supervision during service provision, availability of personal protective equipment (PPE), utilization of telephones or virtual visits for non-urgent cases, multi-month provision of antiretroviral (ARV) medication with phone follow up, mass masking of workers and clients with respiratory symptoms, prioritizing testing for those most in need, ensuring availability of ART and condoms, and counselling |
| Beima-Sofie et al. (2020) | Document insights from clinics leaders relating to challenges and recommendations in the following key areas: program operations, counseling services, testing services, pharmacy services, and other essential services | Seattle | United States of America | Notes from the field | Notes from the field | People living with or at risk of acquiring HIV | 2020 | Challenges and recommendations in the following key areas: program operations, counseling services, testing services, pharmacy services, and other essential services | Providing telemedicine/phone visits for clients with stable virval load, increasing outreach support, linking clients to online support groups, enabling flexibility in testing requirements for drug refills and non-urgent medical conditions, and tailoring pharmacy services to individuals’ risk and medical urgency (e.g., longer drug dispensing, wave testing for drug refills) |
| Boyd et al. (2021) | Describe the adapted strategies and their implementation and demonstrate the continued progress of the ART Surge in providing and expanding access to HIV services during the COVID-19 pandemic | National | Nigeria | Report | De-identified health facilities and community-based HIV service data | People living with HIV | February – September 2020 | Adapted strategies and their implementation | An intensified focus on community‑based, rather than facility‑based, HIV case finding; immediate initiation of newly‑diagnosed PLHIV on 3‑month ART starter packs (first ART dispense of 3 months of ART); expansion of ART distribution through community refill sites; and broadened access to multi‑month dispensing (MMD) (3–6 months ART) among PLHIV established in care. |
| Enane et al. (2022) | Assess healthcare worker (HCW) perspectives on impacts of the COVID- 19 pandemic on adolescent HIV care delivery and engagement | Western Kenya | Kenya | Qualitative study | Interviews | Health workers | January – March 2021 | Perspectives on impacts of the COVID- 19 pandemic on adolescent HIV care delivery and engagement | Expanded roles and leveraging available resources to support adolescent retention and access to care by providing multi- month ART refills, providing refills at local dispensaries as needed, performing lab tests at local facilities as needed, frequent follow- up by phone, and providing remote phone support for care needs, ART refills, and labs |
| Fisher et al. (2022) | Identify programmatic changes in 1) the number of reported positive HIV test results, 2) the number of PLHIV receiving ART, and 3) the percentage of PLHIV receiving ART with suppressed HIV viral load** during 2020 to assess change before and during the first year of the COVID-19 pandemic | 41 PEPFAR supported countries | Multinational | Report | Programmatic data | People living with HIV | 2020 | 1) the number of reported positive HIV test results, 2) the number of PLHIV receiving ART, and 3) the percentage of PLHIV receiving ART with suppressed HIV viral load** during 2020 to assess change before and during the first year of the COVID-19 pandemic | Reducing or streamlining facility attendance through strategies such as enhancing index testing (offering of testing to the biologic children and partners of people living with HIV) and community- and home-based testing; treatment delivery approaches; and improvements in data use through monitoring activities, systems, and data quality checks. |
| Hegarty, Handayani, Nanwani, and Praptoraharjo (2021) | Documented the impact of the COVID-19 pandemic on HIV outreach programmes for men who have sex with men | Jakarta | Indonesia | Qualitative study | Interviews and focus group discussions with outreach workers | Men who have sex with men | July-September 2020 | Impact of the COVID-19 pandemic on HIV outreach programme | Outreach workers shifted activities to ‘virtual outreach’ or online to gain visibility; (2) build and maintain relationships; (3) provide HIV and safe-sex information; (4) follow up with clients; (5) refer clients to healthcare providers for testing/treatment |
| Hoke et al. (2021) | To provide accounts of when and how decisions were taken to support ART home delivery during COVID-19 | Multinational | Multinational (Indonesia, Laos, Nepal, and Nigeria) | Report | Field Action Report | Not identified | May-November 2020 (Nigeria);  April-November 2020 (Indonesia); march-September 2020 (Nepal); and May-September 2020 (Laos) | Programmatic information about home delivery implementation, and lesson learnt for successful expansion of ART home delivery | Nepal, Laos, and Nigeria relied heavily on existing networks of community health workers for ART home delivery. Indonesia mainly used private sector courier service when performing ART home delivery. Lesson learnt included the importance of rapidly engaging stakeholders, investing in differentiated service delivery, protecting outreach workers and clients, and ensuring intervention sustainability. |
| Hong et al. (2020) | Rapid coordination of the national HIV treatment program with the national COVID-19 response | Ministry of Health and Social Services | Namibia | Report | Report | Not identified | March – April 2020 | Frequency of patient contact with the health care system and reduce burden on facilities | Assessing facility readiness for screening and triaging patients, four months multi-month dispensing (MMD) of ART was initiated, and community ART dispensing points were expanded through  1) newly established community-based ART points, 2) primary health care outreach points, 3) community adherence groups, 4) mobile vans, and 5) home delivery. Fast-tracking refills of ART without having patients enter the facility |
| Kay and Musgrove (2020) | Document adaptations during COVID-19 to continue providing HIV services | Birmingham, Alabama | United States of America | Report | Field notes | Not identified | March 2020 | Adaptations during COVID-19 to continue providing HIV services | Counselling services, legal services, and HIV and LGBTQ support group meetings have been conducted either through telephone or secure videoconferencing. Paperwork is completed using email or over the phone. Nutritional services, transportation vouchers, medical items, pet food, and personal care items are provided through curbside service, allowing clients to remain in their cars while BAO staff load any of the previously mentioned items into their vehicles. Case managers can complete any needed assessments over the phone and, if paperwork must be exchanged, a designated BAO employee is in charge of transferring paperwork from clients’ cars |
| McGinnis et al. (2021) | Clinic visit vs telephone/video (virtual) ART medication refill | Veterans Affairs | United Sates of America | Cohort study | Cohort | People with HIV enrolled in the Veterans Aging Cohort Study | March 2019- February 2020; March 2020-February 2021 | Percentage of in-person vs telephone/video (virtual) ART medication refill | VA increased the use of virtual visits and longer ART refills. ART refills were increased to more than three months, increasing from 39% in 2019 to 51% in 2020. Virtual visits also increased from 27% in 2019 to 64% in 2020. |
| Mpofu et al. (2021) | Assess willingness and capacity to dispense ART to people living with HIV enrolled in the Botswana national ART program | 10 high volume public facilities in Gaborone | Botswana | Mixed methods (survey and interviews) | Interviews with people with HIV and online survey with private pharmacies | People with HIV | July – August 2020 | Acceptability and reach of ART home delivery | Use of private pharmacies to distribute ART. 62% of private pharmacies showed willingness to provide ART. 61% of people enrolled in ART home delivery were reached. |
| Nyunt et al. (2021) | Reports on the Contingency Plan for HIV and its implementation in the first few months as an example on how low- and middle-income countries can work to provide essential services to People Living with HIV and Key Populations. | National level | Myanmar | Programme-based cross-sectional descriptive study | Routinely collected data from MoHS data system | People living with HIV and key populations | Comparing first six months of 2019 and 2020 | Contingency Plan for HIV and its implementation in the first few months as an example on how low- and middle-income countries can work to provide essential services to People Living with HIV and Key Populations | Introduction of HIV self-testing was initiated, ART patients were receiving 6 months ARV dispensing,  Mitigating the potential supply chain disruption to guarantee uninterrupted supply of ARV medicines and other commodities; preponing international shipping; expanding the storage capacity of warehouses; and the flow of the medicines to support the decisions on long-term take-home doses or multi-month dispensing (MMD) of ART.  Community involvement, including engaging community Networks, National and International NGOs from the beginning of the crises, considering their important role in the National Response to HIV.  The HIV-related workforce was re-organized by their schedule so as not to overwhelm the health facilities; the organization of a back-up workforce in case personnel and volunteers got infected by COVID-19 and were secluded from their workplace; the intense reduction of physical meetings, trainings or workshops; the expansion of online communication tools and strategy to guarantee participation; the extension of this approach to peer educators and community volunteers and the natural necessary postponement of activities that were not critical. |
| Phiri et al. (2022) | describe adaptations to the Yathu Yathu intervention in response to the COVID19 epidemic, and implications on uptake of HIV testing services among adolescent and young people | Lusaka | Zambia | Mixed methos | Routinely collected PPC data and qualitative data | Adolescent and young people | 2020 | Uptake of HIV testing services before and after adaptations in response to COVID-19 | Use of WhatsApp groups and Facebook page as platforms |
| Pollard et al. (2021) | Assess the acceptability of HIV services adaptations and impact of the pandemic among key populations | Telangana and Maharashtra | India | Qualitative study | Focus group discussions | Men who have sex with men, female sex workers and transgender women | November - December 2020 | The impact of COVID-19 on access to HIV services among key populations | Community-based organisation arranging transportation to facilitate access to testing, making multi-month dispensing of ART available to all people with HIV, expanding home deliveries of ART, allowing ART pickup from any public ART centre in the country rather than the centre where clients are registered, |
| Quilantang, Bermudez, and Operario (2020) | Describe strategies use for HIV services implementation during COVID-19 | Philippines | Philippines | Notes from the Field | Notes from the field | People living with or at risk of acquiring HIV | 2020 | strategies use for HIV services implementation during COVID-19 | creating and maintaining online platforms, and adapting telemedicine for clinical consultations and counselling. some CBOs have designated “skeletal workforces” comprising a subset of core staff/volunteers who continue to deliver basic services to PLHIV and members of high-risk groups, even during the most acute phases of community quarantine implementation and physical distancing measures. CBOs have started delivering ART medications to PLHIV at designated drop-off points. the development of mobile applications that include a map of ARV medication distribution points |
| Samudyatha, Kosambiya, and Rathore (2022) | Document the process, strengths and challenges of adopting multi-month dispensing | Surat city of South Gujarat | India | Mixed method | Desk review of programmatic data | People living with HIV and HIV service provider staff | March – May 2020 | Process of MMD and Community Dispensation of ART during the lock-down period,  and the strengths and challenges of the MMD and Community Dispensation Strategies adopted during the lock-down period | Multi Months Dispensation (MMD) through ART centres and Community Dispensation of ART through the various Targeted Interventions (TI) and Community Based Organizations (CBO). Home delivery through targeted interventions/ Community Based Organizations, people living with HIV networks) |
| Sun et al. (2021) | Explore barriers to ART maintenance and solutions to ART interruption when stringent COVID-19 control measures were implemented in China, from the perspective of people living with HIV and relevant key stakeholders | Various regions | China | Qualitative study | Semi-structured interviews | People with HIV, community-based organisations, staff from disease control and prevention centres, HIV doctors and nurses, drug vendors | February 2020 | Challenges and responses relevant to ART continuity during COVID-19 | Community based organisations helped people with HIV maintain access to ART in five ways, including (a) coordination to refill ART from local CDC clinics or hospitals, (b) delivery of ART by mail, (c) privacy protection measures, (d) mental health counselling, and (e) providing connections to alternative sources of ART. Drug vendors contributed to ART maintenance by selling out-of-pocket ART. |
| Yelverton, Qiao, Weissman, Olatosi, and Li (2021) | understand telehealth utilization for HIV care services in South Carolina (SC), identify barriers to telehealth during COVID-19, and investigate strategies to facilitate remote HIV care delivery | South Carolina | United States of America | Qualitative study | Interviews | management personnel of an academic medical center, local ASOs, and the SC state public health agency | July 2020 | Telehealth utilization for HIV care services in South Carolina (SC), identify barriers to telehealth during COVID-19, and investigate strategies to facilitate remote HIV care delivery | Utilizations of telehealth were diverse in delivering medical and non-medical HIV care services, cell phone distribution, bureaucracy and process adjustments, staff education, client empowerment and technology use guidance, reimbursement changes, guidance for remote services |
| Zakumumpa, Tumwine, Milliam, and Spicer (2021) | Explore health-system resilience at the sub-national level in Uganda with regard to strategies for dispensing antiretrovirals during Covid-19 lockdown | Eight districts in Eastern and Western Uganda | Uganda | Qualitative case-study | Interviews and focus group discussions | District health team leaders, ART clinic managers, representatives of President’s Emergency Plan for AIDS Relief (PEPFAR) implementing organization, and people with HIV | June-September 2020 | Health-system resilience at the sub-national level in Uganda with regard to strategies for dispensing antiretrovirals during Covid-19 lockdown | Strategies for distribution of ART included: accelerating home-based delivery of ART; extending multi-month dispensing from three to six months; using Community Drug Distribution Points model for ART refill pick-ups at outreach sites in the community; increasing reliance on health information systems, including geospatial technologies, to support ART refill distribution in unmapped rural settings. |
